# Progress in treatment of pathological neuropathic pain after spinal cord injury

**DOI:** 10.3389/fneur.2024.1430288

**Published:** 2024-11-11

**Authors:** Jian Li, Wenqing Kang, Xi Wang, Fang Pan

**Affiliations:** ^1^Department of Orthopedics, Central Hospital Affiliated to Shandong First Medical University, Jinan, Shandong, China; ^2^Xuanwu Jinan Hospital, Jinan, China; ^3^Department of Neurology, Yidu Central Hospital of Weifang, Weifang, Shandong, China; ^4^Department of rehabilitation, Shandong Rehabilitation Hospital, Jinan, Shandong, China

**Keywords:** spinal cord injury, neuropathic pain, glial cell, electroacupuncture, spinal cord stimulation

## Abstract

Pathological neuropathic pain is a common complication following spinal cord injury. Due to its high incidence, prolonged duration, tenacity, and limited therapeutic efficacy, it has garnered increasing attention from both basic researchers and clinicians. The pathogenesis of neuropathic pain after spinal cord injury is multifaceted, involving factors such as structural and functional alterations of the central nervous system, pain signal transduction, and inflammatory effects, posing significant challenges to clinical management. Currently, drugs commonly employed in treating spinal cord injury induced neuropathic pain include analgesics, anticonvulsants, antidepressants, and antiepileptics. However, a subset of patients often experiences suboptimal therapeutic responses or severe adverse reactions. Therefore, emerging treatments are emphasizing a combination of pharmacological and non-pharmacological approaches to enhance neuropathic pain management. We provide a comprehensive review of past literature, which aims to aim both the mechanisms and clinical interventions for pathological neuropathic pain following spinal cord injury, offering novel insights for basic science research and clinical practice in spinal cord injury treatment.

## 1 Introduction

Traumatic Spinal cord injury (SCI) is a prevalent critical clinical condition, with an estimated incidence ranging from 15 to 40 cases per million, attributed to various factors such as traffic accidents, falls from high altitudes, and violent impacts (1, 2). Patients afflicted with spinal cord impairment often experience subsequent sensory and motor dysfunctions, leading to a cascade of complications including secondary pulmonary infections, deep venous thrombosis, urinary retention, pressure ulcers, pain, and psychological distress (3). Neuropathic pain (NP) stands out as one of the most prevalent complications following SCI, affecting approximately 53 to 80% of individuals (4). This condition manifests as spontaneous pain, paresthesia, or hyperalgesia below the level of injury, predominantly in the lower limbs, with pain intensity typically higher at night than during the day (5, 6). Spontaneous pain can be further categorized into continuous and intermittent forms; the former is characterized by sensations of burning or squeezing, while the latter presents as electric shock-like pain or tingling sensations. Hyperalgesia involves heightened responses to noxious stimuli, while allodynia refers to various abnormally evoked responses to innocuous stimuli (7, 8). These symptoms significantly diminish patients’ quality of life and impose substantial personal and socioeconomic burdens (4, 9, 10). Moreover, NP can precipitate anxiety disorders, depression, substance abuse, and other mental health conditions, and in severe cases, suicidal ideation (11, 12).

The pathogenesis of NP following SCI is intricate and arises from a combination of multiple pathological reactions (13). The development and maintenance of pain involve the peripheral nervous system, the spinal cord, and the higher central nervous system structures above the spinal cord. Due to the complex pathogenesis of neuralgia after SCI, its treatment has always posed a significant challenge in clinical practice. Currently, drugs primarily used for NP treatment are opioid analgesics, anticonvulsants (14), such as Tramadol (15), gabapentin (16, 17), pregabalin (18), lamotrigine (19), and amitriptyline (20), which aim to alleviate pain. However, many patients remain dissatisfied with pain relief (21). Simultaneously, these medications are associated with numerous side effects, including fever, nausea, dizziness, rash, weakness, drowsiness, and other psychiatric disorders (14, 21, 22). Additionally, various other therapeutic strategies exist, such as natural compounds (23, 24), electroacupuncture stimulation (25), repetitive transcranial magnetic stimulation therapy (26), transcranial direct current stimulation (27), and spinal cord stimulation (28). In this review, we discuss and analyze the mechanisms and therapeutic measures of NP after SCI in hopes of aiding for better treatment.

## 2 Mechanisms of pathologic neuropathic pain following SCI

**Figure 1:**
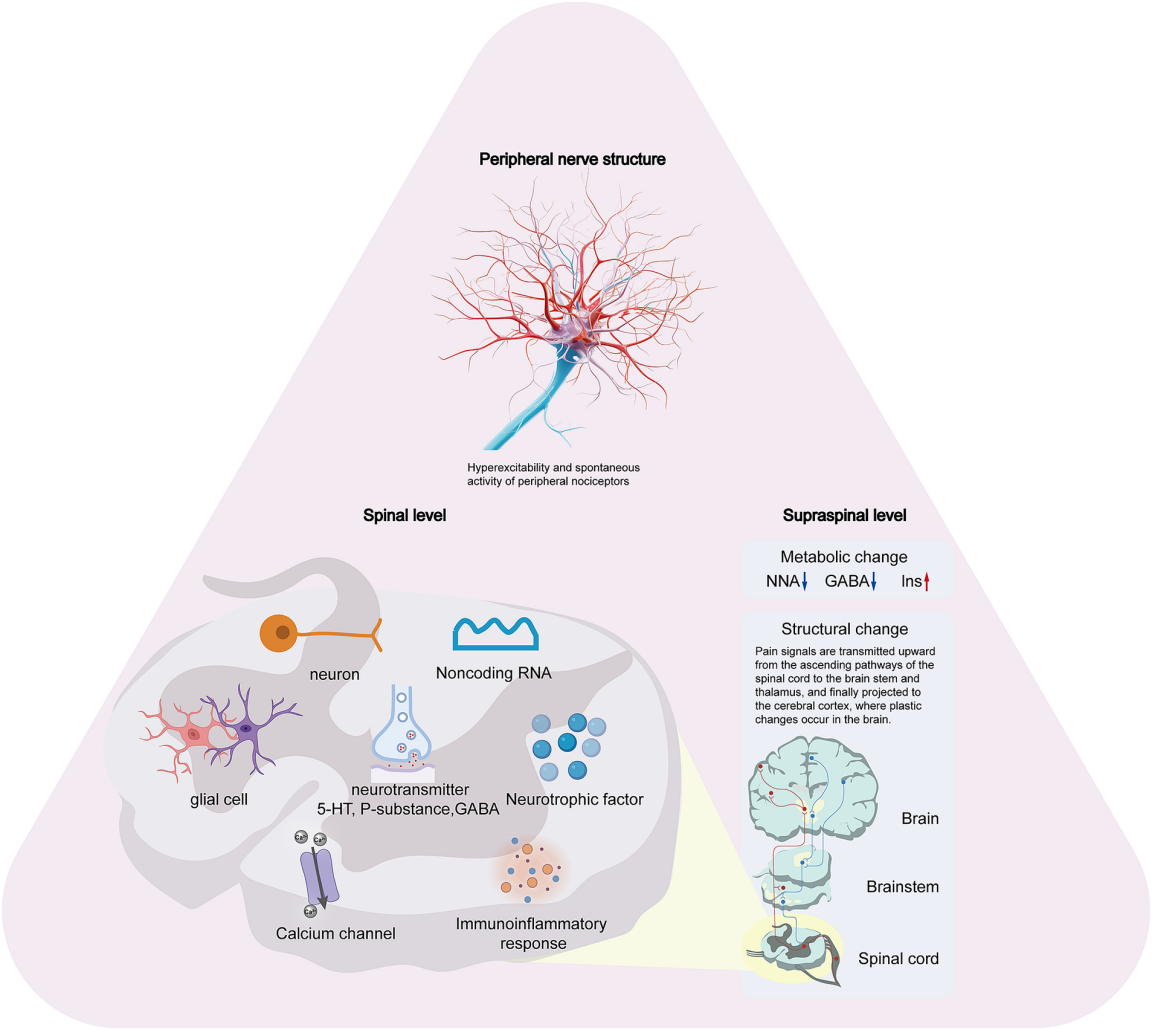
The schematic representation of the pathophysiology of NP following SCI.

NP following SCI is characterized by alterations in normal sensory signals at various levels, including peripheral structures, the spinal cord, and supraspinal regions. These changes occur over weeks or months, leading to an amplification of nociceptive information and resulting in central sensitization of pain perception. NP following SCI is generally characterized by widespread and multifaceted sensory loss and/or chronic pain (29, 30). Pain was observed not only at the site of the injury but also in regions below the level of the injury, and it did not diminish over time. The underlying mechanisms are complex. To elucidate the pathophysiology of neuropathic pain following SCI, this section will be divided into three parts: the peripheral nervous system, the spinal cord itself, and the supraspinal structures. Figure 1 illustrates the schematic representation of the pathophysiology of NP following SCI.

### 2.1 Peripheral level

Current mechanistic studies on NP after SCI have primarily focused on neuronal changes within pain pathways at both spinal and supraspinal levels, particularly those associated with inflammation and glial activation. However, chronic hyperexcitability also occurs in primary nociceptors following SCI, leading to altered function and spontaneous activity of peripheral receptors throughout the neural pathway (31). Neuronal alterations in spinal and supraspinal pain pathways affect central processes in primary sensory neurons, triggering hyperexcitable states and spontaneous activity in nociceptors, which consequently drive hypersensitivity and pain (31). Increasing preclinical evidence indicates that mitigating heightened activity in primary sensory neurons by selectively targeting receptors in peripheral nervous system effectively alleviates peripheral nervous system pain (32). Ritter et al. (33) reported on the downregulation of Kv3.4 potassium channels in dorsal root ganglion neurons post-SCI, highlighting its dysregulation in SCI-NP models. This downregulation contributes to the hyperexcitability of nociceptors, forming the basis for persistent pain after SCI. Yang et al. (34) showed that downregulation of Nav1.8 channels was able to reduce SCI-induced spontaneous nociceptor activity, thereby further reducing SCI-NP. Two weeks post-cervical hemicontusion injury, contralateral nociceptor hyperexcitability was observed, attributed to diminished Kv3.4 channel membrane expression (35). In 2017, the group elucidated in a novel publication that spinal cord injury (SCI)-induced Kv3.4 channel dysfunction is mediated through the inhibition of the calcineurin phosphatase (36).

### 2.2 Spinal level

After a SCI, numerous pathological changes occur within the spinal cord, leading to a heightened focus on NP pathogenesis research. Key areas of study include alterations in neuronal excitability (37), glial cell activation (38, 39), upregulation of calcium channel expression (7, 40, 41), immune-inflammatory responses (42), disrupted neurotransmitter secretion (43), imbalances in neurotrophic factors (44), and the roles of non-coding RNAs (2).

#### 2.2.1 Neuronal and glial cells in post-spinal cord injury neuropathic pain

Neuronal hyperactivity, characterized by heightened spontaneous excitability or abnormal increases in neuronal activity in response to thermal, chemical, and mechanical stimuli, is a notable phenomenon. In the context of SCI, ischemia and upregulated neurotrophic factor levels contribute to the structural atrophy of the spinal cord and synaptic circuit alterations. These changes induce spontaneous and secondary hyperexcitability in spinal cord sensory neurons, leading to a decreased threshold potential, enlarged receptive fields, and heightened nociception to similar stimuli (37, 45–47). The hallmark of this state is the shift of the resting membrane potential toward less negative values, potentially precipitating spontaneous depolarization and the subsequent activation of neurons, a phenomenon termed “central sensitization.”

Glial cells, including astrocytes, microglia, and oligodendrocytes, are widespread in the nervous system. SCI triggers the activation of astrocytes and microglia, resulting in a notable increase in cell size and the thickening of their processes (48). This activation leads to the release of bioactive substances and cytokines, which heighten the sensitivity of spinal dorsal horn nerves to pain sensation and contribute to the maintenance of pathological pain (38, 39, 49, 50). The time course of astrocyte activation appears to coincide with both the transition from acute to chronic pain and the maintenance phase of chronic pain (51). Astrocyte activation following SCI is dependent on the secretion of several proteins, including connexin 43, calbindin S100B, and aquaporin-4. In numerous NP models, there is a notable increase in the number of gap junction channels and an upregulation of connexin 43 (Cx43) expression (52). This upregulation contributes to the secretion of various cytokines involved in NP development. Cx43 is predominantly expressed in astrocytes, and gap junctions formed by Cx43 play a critical role in the pathogenesis of NP following SCI. Post-injury, there is a substantial increase in gap junction channels both between neurons and glial cells, as well as among glial cells themselves. Additionally, glial cells exhibit enhanced sensitivity to pain mediators such as ATP. Prolonged exposure to ATP leads to the release of a cascade of cytokines from these cells, which further activates glial cells and exacerbates neuronal damage (52). Research indicates that Cx43 is rapidly and persistently upregulated in astrocytes following SCI. Furthermore, the use of Cx43 inhibitors has been linked to relief from NP (52, 53). S100β, a calcium-binding protein of the EF-hand family, is predominantly expressed by astrocytes within the central nervous system. Its production and secretion are markedly elevated in reactive astrocytes. At high extracellular concentrations, S100β exhibits neurotoxic effects and its secretion into the extracellular space further stimulates astrocyte activation, leading to S100β autocrine signaling (54). Inhibiting S100β production by astrocytes may provide an effective strategy to suppress astrocyte activation. In a rat model of incomplete SCI, the administration of an S100β inhibitor was found to alleviate NP following SCI. Histological analyses support the conclusion that inhibiting S100β production and astrocytic activation contributes to the reduction of NP. Moreover, there is a strong correlation between the intensity of S100β expression at the injury sites and the severity of NP (55). Aquaporins are a family of small, integral membrane proteins, with Aquaporin-4 (AQP-4) being a prominent member and one of the most extensively studied aquaporins (56). Evidence from both *in vitro* and *in vivo* studies suggests that AQP-4 plays a critical role in astrocyte activation. In wound healing and scratch assays, impaired migration was observed in astrocyte cultures derived from AQP-4 deficient mice compared to wild-type mice, with no significant differences noted among the wild-type samples (57). Auguste et al. employed an adult mouse astrocyte migration assay to investigate the involvement of AQP-4 in astrocyte migration *in vivo*. Their findings revealed that AQP-4 +/+ astrocytes exhibited preferential migration toward the wound site, whereas AQP-4 −/− astrocytes demonstrated significantly reduced migratory ability (56, 58). Additionally, Yu et al. utilized a rat T13 spinal cord hemi-section model and found that AQP-4 expression was significantly upregulated in in L4/5 spinal cord segments, primarily in astrocytes within the spinal dorsal horn (SDH).

Microglia play both deleterious and protective roles in NP following SCI (59). Activation of microglia results in the release of proinflammatory cytokines and chemokines that enhance neuronal reactivity, thereby exacerbating pain (60–62). The formation of a glial scar is a typical response to SCI. This scar comprises astrocytic processes and a significant quantity of glial fibers. Although the mechanical strength of a glial scar is inferior to that of a collagenous scar, it plays a crucial role in providing structural support to the injured tissue and in preventing further damage (63). However, glial scars can also contribute to the emergence of NP, with microglial activation considered necessary for glial scarring (64–66). Additionally, microglia have functions such as removing cell debris, tissue repair, promoting the release of anti-inflammatory cytokines, and aiding axonal regeneration, which can have a positive impact on pathological NP after SCI (67–69). The role of oligodendrocytes in the pathogenesis of NP has been less explored, but given their abundance as a type of glia, their role cannot be disregarded. Many multiple sclerosis patients experience pain alongside oligodendrocyte loss, suggesting a potential interaction between oligodendrocyte damage and human pain (70). Furthermore, studies have shown that oligodendrocyte ablation using diphtheria toxin in adult mice leads to the development of NP behaviors (71). As research on oligodendrocytes continues, the involvement of activated glial cells in pain transmission after SCI will be further elucidated.

#### 2.2.2 Calcium channel in post-spinal cord injury neuropathic pain

Voltage-gated calcium channels depolarize the cell membrane, allowing calcium to enter the cell. This process has various physiological effects, including secretion, contraction, neurotransmission, and gene expression (72). The alpha-2-delta-1 (Cavaα2δ-1) subunit protein plays a crucial role in the functional assembly of voltage-gated calcium channels, regulating calcium channel current density and synaptogenesis (73–75). Experimental evidence has shown that excessive up-regulation of the calcium channel Cavaα2δ-1 subunit protein in the spinal dorsal horn leads to the development of tactile allodynia in Peripheral nerve injury model (76). Conversely, blocking the up-regulation of the Cavaα2δ-1 subunit protein, such as through knockdown of Cavaα2δ-1, Cavaα2δ-1 antisense oligodeoxynucleotide, or drugs inhibiting Cavaα2δ-1 subunit protein, can prevent and treat the development of pathological NP (7, 77, 78).

#### 2.2.3 Immuno-inflammatory reaction in post-spinal cord injury neuropathic pain

The immune-inflammatory responses triggered by SCI play a crucial role in the development of NP. Pro-inflammatory factors exacerbate local tissue damage, while anti-inflammatory factors aid in repair, depending on the timing and activation state of immune cells (79–82). After SCI, the permeability of the blood-spinal cord barrier (BSCB) increases, allowing macrophages, granulocytes, and lymphocytes from the bloodstream to infiltrate the injured spinal cord (83). These cells, along with other inflammatory factors, induce inflammatory responses in the tissues surrounding the injury site, leading to neuronal apoptosis, scarring, and dysfunction of peripheral nerves within the injured spinal cord (84–87). In the context of SCI, M1 macrophages exert detrimental effects on nerves, whereas M2 macrophages promote neuronal and axonal regeneration and mitigate local inflammatory responses (88, 89). Following SCI, M1 macrophages are rapidly and persistently upregulated, while M2 macrophages are expressed more slowly and transiently. The imbalance in M1/M2 macrophage expression results in a robust inflammatory response that contributes to pain induction (90).

Moreover, the migration of T and B lymphocytes to the injury site after SCI initiates a multifaceted adaptive immune response. This response is characterized by increased expression of pro-inflammatory cytokines such as IL-1*α*, IL-1β, and TNF-α, which transmit noxious information to the central nervous system and exacerbate secondary injury in patients with acute or chronic SCI (91). These pro-inflammatory factors may sensitize injured spinal dorsal horn neurons by promoting the release of excitatory amino acids and substance P, thereby inducing pain (92, 93).

#### 2.2.4 Neurotransmitter in post-spinal cord injury neuropathic pain

Serotonin (5-HT) is an endogenous neurotransmitter widely distributed throughout the nervous system, exerting dual effects of both causing and relieving pain (94, 95). While 5-HT1 and 5-HT2 receptor subtypes generally have analgesic effects, models of persistent pain suggest that activation of the 5-HT3 receptor plays a role in sustaining pain (94). In the central nervous system, serotonergic neurons are primarily located in the raphe nucleus of the brainstem and play a crucial role in the descending analgesic system; intrathecal injection of 5-HT demonstrates a significant analgesic effect after SCI (96). Following SCI, 5-HT within the peripheral nervous system can transmit nociceptive signals either directly through second messengers or indirectly by modulating ion channel activity (97). Glutamate (Glu) in the central nervous system is implicated in the transmission of nociceptive information and neurotoxicity, closely associated with the onset of pain following SCI (98). Gamma-aminobutyric acid (GABA) serves as an inhibitory neurotransmitter, whose expression decreases post-SCI, consequently disrupting the “GABA-glutamate-glutamine cycle” *in vivo* (99, 100). Studies have shown that the administration of GABA receptor agonists significantly alleviates pain in rats with SCI (99). Substance P, a neuropeptide closely linked to pain, also participates in the regulation of NP following SCI. Inhibiting the release of substance P delays the onset of NP by 1 to 4 days (101). Additionally, substance P-fragment 1–7, a metabolite of substance P, demonstrates significant pain-alleviating effects when administered peripherally to alleviate SCI-induced pain (102).

#### 2.2.5 Neurotrophic factors in post-spinal cord injury neuropathic pain

The neurotrophic family includes neurotrophin-3 (NT-3), ciliary neurotrophic factor (CNTF), basic fibroblast growth factor (bFGF), insulin-like growth factor (IGF), glial cell-derived neurotrophic factor (GDNF), and brain-derived neurotrophic factor (BDNF). These factors are crucial for promoting the survival, proliferation, and axonal regeneration of various cell types following SCI. Neurotrophin-3 (NT3) is a potent neurotrophic factor that plays a crucial role in neuronal regeneration and functional recovery following SCI. Besides sustaining the survival of sympathetic neurons, sensory neurons, basal forebrain cholinergic neurons, and motor neurons, NT3 also supports the differentiation of dopaminergic neurons and promotes the sprouting of lateral branches of the corticospinal tract (CST) (103). However, spinal cord function markedly improves with elevated NT3 concentrations (104). Ciliary neurotrophic factor (CNTF), a polypeptide, activates signaling cascades including JAK/STAT, MAPK, ERK1/2, AMPK, mTOR, and AKT via its receptors (105–108). CNTF exerts pivotal roles in neuronal development and nervous system homeostasis by enhancing survival and differentiation in sensory, sympathetic, and motor neurons through the modulation of gene expression (109). Fibroblast growth factors (FGFs) are classified into acidic FGF (aFGF) and basic FGF (bFGF) based on their isoelectric properties. Both aFGF (acidic Fgf1) and bFGF can enhance the regeneration of spinal cord and dorsal root ganglion neurons in both human and animal models following SCI (110–112). aFGF exhibits strong neurotrophic effects and promotes neuronal growth (113). The mechanism of action of bFGF includes inhibiting apoptosis and c-fos gene expression at the injury site, stabilizing calcium and magnesium ion levels to prevent toxicity, regulating glial cell responses, and reducing glial scar formation (103). Glial cell line-derived neurotrophic factor (GDNF), a member of the transforming growth factor beta (TGF-*β*) superfamily, exhibits strong neurotrophic effects on motor, sensory, and dopaminergic neurons. It has been shown to effectively stimulate axonal regeneration and promote myelin repair in central nervous system (CNS) injuries (114). Transplantation of lentivirus-mediated GDNF-secreting cells into the SCI site significantly increased nerve fiber density at the injury site and improved motor function recovery (115). The truncated isoform of the tyrosine receptor kinase (BTrkB), a primary receptor for brain-derived neurotrophic factor (BDNF), mediates BDNF signaling through various classical pathways (44, 116, 117). BDNF binds to the tropomyosin receptor kinase (Trk) B receptor, promoting the development, differentiation, and regeneration of sensory neurons, cholinergic neurons, dopaminergic neurons, and GABAergic neurons (114, 118). Additionally, BDNF facilitates myelination, regulates synaptic plasticity, and influences synaptic transmission (119). Notably, BDNF, as a member of the neurotrophic family, also modulates NP following SCI (44, 120, 121). Post-SCI upregulation of BDNF in the spinal dorsal horn is implicated in hyperalgesia and tactile allodynia, with TrkB-specific knockdown in mice markedly attenuating pain responses (44).

#### 2.2.6 Non-coding RNAs in post-spinal cord injury neuropathic pain

Noncoding RNAs (ncRNAs) typically do not encode proteins but rather regulate protein expression and numerous cellular, biochemical, and physiological processes. They are generally classified into micro RNAs (miRNAs), circular RNAs (circRNAs), and long ncRNAs (lncRNAs) (2). MiRNAs, composed of approximately 22 nucleotides, are involved in secondary injury and repair processes following SCI. For example, reduced expression of miRNA-139-5p in the spinal cord of mice post-SCI was associated with pain hypersensitivity, whereas intrathecal administration of miRNA-139-5p agonist mitigated pain hypersensitivity, enhanced survival of damaged spinal cord neurons, and promoted motor function recovery (122). Moreover, miRNA-139-5p was found to reduce pain sensitivity and facilitate functional recovery in SCI mice by targeting 20-like kinase 1 in mammalian sterile lines (122). Additionally, intrathecal injection of miRNA-132-3p mimics in rats mimicked hyperalgesia attributed to spinal *α*-aminomethyloxazolopropionate receptor 1 expression, implicating miRNA-132-3p in pain information processing (123). In a study utilizing peripheral blood samples from SCI patients for sequencing, two lncRNAs (LINC01119 and LINC02447) were directly implicated in the NP pathway (124). Zhao et al. (125) discovered a conserved lncRNA, Kcna2 antisense RNA, correlated with Kcna2 in rat DRG sensory neurons, which is significantly upregulated by peripheral nerve injury, resulting in Kcna2 repression and neuropathic pain onset. Conversely, inhibition of Kcna2 antisense RNA expression effectively reverses the neural injury-induced downregulation of Kcna2 in the DRG, thereby mitigating both the development and maintenance of neuropathic pain. This suggests a potential significant role for these lncRNAs as biomarkers in SCI-induced NP pathways. Circular RNAs, a type of abundant lncRNAs characterized by a closed continuous loop structure, exhibit high stability. These circRNAs, shown to be neuron-specific, modulate miRNA expression during NP pathology (2). The mechanisms and roles of non-coding RNAs in regulating NP after SCI are still in early stages and necessitate further in-depth investigation in the future.

### 2.3 Supraspinal level

The spinal cord, as a central component of the nervous system, not only causes localized pathological changes after injury but also transmits pain signals through its ascending pathways to the brainstem and thalamus, eventually projecting to the cerebral cortex. Following SCI, bidirectional signal transduction between the spinal cord and cerebral cortex is disrupted. This destruction of sensory and motor conduction pathways leads to plastic changes in the structure of the cerebral cortex. Such remodeling affects not only sensory and motor functions but also the regulation of nociceptive information. In the early stages of SCI, atrophy occurs in the primary sensory cortex (S1) and primary motor cortex (M1), with the degree of atrophy correlating positively with the severity of the injury (126). Functional magnetic resonance imaging (fMRI) studies have confirmed dynamic reorganization in the sensory and motor cortices following SCI in patients with complete cervical SCI, revealing decreased functional connectivity between these regions and highlighting the plastic changes in the brain after SCI (127). Patients experiencing pain post-SCI exhibit reduced gray matter volume in the paracentral lobule of the S1 region. Incomplete sensory afferent and efferent information may lead to maladaptive remodeling of the S1 region, which can contribute to pain development. Additionally, a robust correlation exists between the extent of S1 region reorganization post-SCI and the severity of persistent neuropathic pain (128). The subcortical thalamus, serving as a relay station for sensory information, receives various types of sensory input (excluding olfactory information) from across the body. It plays a crucial role in sensing and regulating nociceptive information and is considered a key site for endogenous pain modulation (129). Seminowicz et al. (130) demonstrated that, 7 days after SCI, functional connectivity between the ventral posterolateral nucleus (VPL) and the S1 region of the thalamus decreased, while connectivity between the S1 region and other nociceptive processing cortical areas (such as the insula and anterior cingulate cortex) increased. By day 14 post-injury, connectivity between the VPL and the contralateral thalamus had increased. The temporal correlation between the enhanced functional connectivity within thalamic and cortical regions and the development of mechanical hyperalgesia in SCI rats suggests that abnormal pain perception after SCI may result from dysregulation in functional connectivity between the thalamus and pain perception cortical regions.

Metabolic changes in the brain after SCI also significantly impact NP. Magnetic resonance spectroscopy (MRS) can detect alterations in brain metabolism in NP patients following SCI, providing further insight into pain mechanisms. Studies have found changes in cingulate metabolism in NP patients post-SCI, including decreased levels of N-acetylaspartate (NAA) and *γ*-aminobutyric acid (GABA), and increased levels of inositol (Ins) (131, 132). These metabolic changes correlate with pain severity, with the NAA/Ins and glutamate (Glu)/Ins ratios significantly affected. Additionally, GABA aggregation has been associated with changes in functional connectivity, with thalamic GABA content negatively correlating with connectivity in thalamocortical tracts. Specifically, greater loss of thalamic GABA corresponds to closer connections between the VPL and other brain regions such as S1, S2, and the Insular lobe (131).

## 3 Pharmacological therapeutic interventions of pathologic neuropathic pain following SCI

### 3.1 Commonly used clinical pharmacological therapeutic interventions for post-spinal cord injury neuropathic pain

Clinically, various drug treatments are available for NP following SCI. However, some patients may experience poor therapeutic outcomes or serious adverse reactions (21, 133). Anticonvulsants are commonly utilized in clinical practice, with gabapentin drugs (pregabalin, gabapentin) being particularly prevalent. These drugs do not directly act on GABA receptors but enhance inhibitory neuronal activity through interactions with N-type voltage-gated calcium channels or indirectly on NMDA receptors. Additionally, they reduce glutamate release and inhibit nociceptive information transmission in chronic constriction injury model (134), but have not been validated in SCI models. Numerous studies have demonstrated the pain-relieving effects of pregabalin and gabapentin in SCI-induced NP, with common adverse effects including mild to moderate transient drowsiness, dizziness, and edema (133). Amitriptyline, a commonly used tricyclic antidepressant in clinical practice, has demonstrated some analgesic efficacy for NP following SCI in randomized controlled trials (135). Tricyclic antidepressants exert their effects through multiple mechanisms, primarily by inhibiting the reuptake of norepinephrine and serotonin, thus inhibiting nociceptive information transmission (136, 137). Common side effects of amitriptyline at an average maximum daily dose of 50 mg include dry mouth, drowsiness, fatigue, constipation, increased cramps, urinary retention, and sweating (133, 138). Anticonvulsants like lamotrigine reduce neuronal hyperexcitability by inhibiting voltage-sensitive sodium channels (Nav) and inhibiting the pathological release of glutamate (19). However, lamotrigine is indicated only for NP caused by incomplete SCI and has side effects such as vertigo, nausea, visual impairment, and rash (19, 139). Opioid analgesics are also clinically utilized to manage neuralgia post-spinal cord injury. Tramadol’s analgesic effect results from weak opioid-like mechanisms and monoaminergic actions, as well as a synergistic effect of the two. A randomized, double-blind, controlled study demonstrated that tramadol reduced pain intensity scores and injury severity in 35 SCI patients after 4 weeks of treatment, significantly alleviating pain below the injury level and improving sleep, although it did not significantly improve depression (15). Therefore, the pursuit of newer, more effective, and safer treatment modalities for post-SCI neuralgia continues.

### 3.2 Natural compound for post-spinal cord injury neuropathic pain

The development of a novel drug entails a substantial investment of time and financial resources (140, 141). Therefore, natural compounds have garnered attention in the treatment of various diseases in recent years due to their minimal side effects and cost-effectiveness (142). Han et al. developed a rat model of thoracic SCI to demonstrate that intrathecal administration of resveratrol effectively alleviated pain post-SCI in rats, possibly by inhibiting neuroinflammation via the JAK2/STAT3 signaling pathway in the lumbar spinal dorsal horns (143). Additionally, several other natural compounds, including salidroside, betulinic acid, and quercetin, have shown promising results in the function recovery of post-spinal cord injury, but more evidence is needed to confirm these role in treating neuropathic pain (144–146).

### 3.3 Antisense oligonucleotides as a prospective therapeutic agent for post-spinal cord injury neuropathic pain

**Figure 2:**
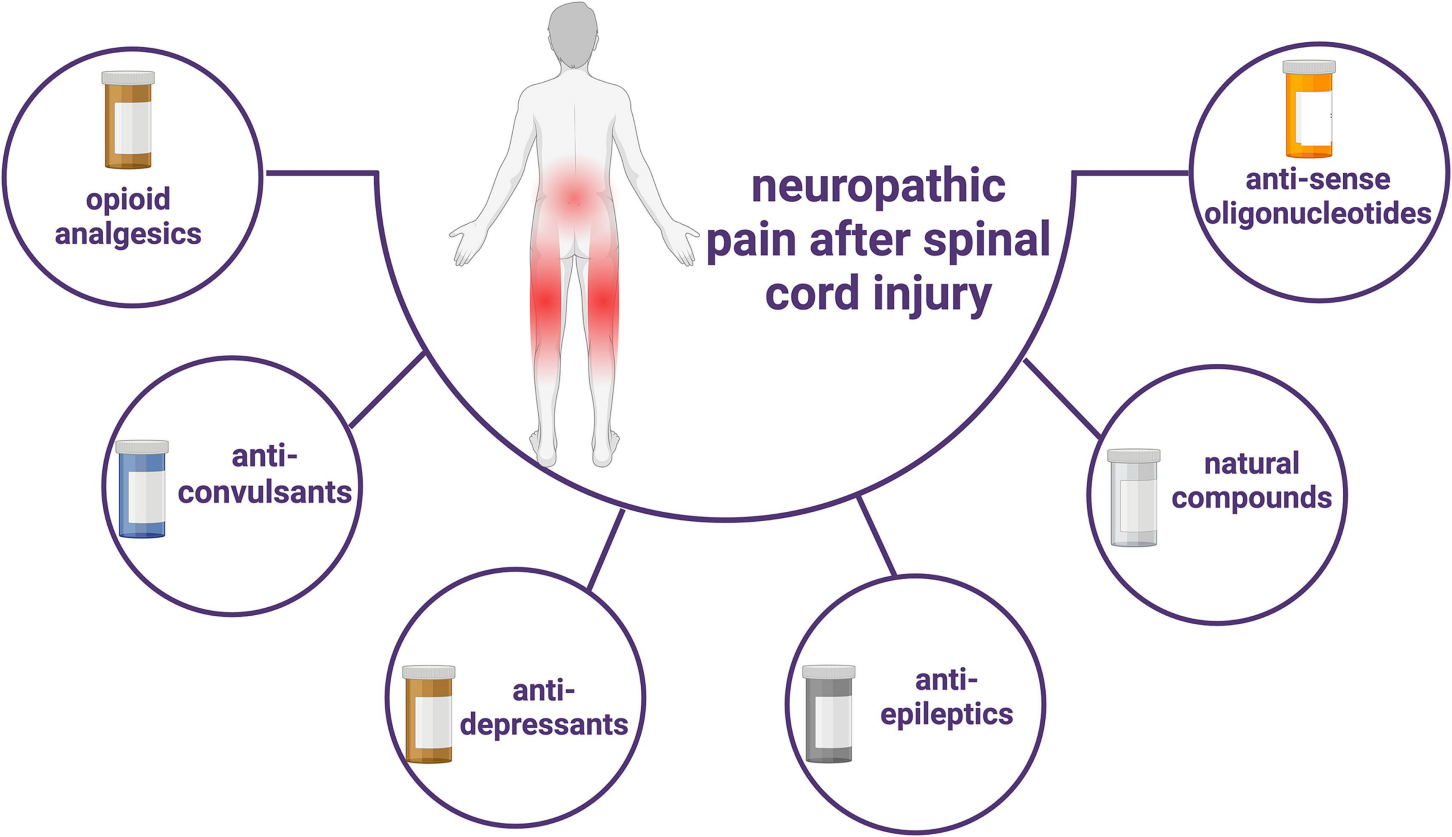
Current types of pharmacological treatments for SCI-induced neuropathic pain.

Antisense oligonucleotides represent a class of drugs designed to modulate expression levels by targeting both coding and non-coding RNAs, showing considerable potential across diverse fields including genetic diseases, cancer, neurodegenerative diseases, and NP (147). These oligonucleotides exhibit specificity based on their sequence, thereby offering the capability to target virtually any known RNA sequence. Their customizable sequences and chemistry render them highly versatile and adaptable (147). This therapeutic approach has garnered approval from the US Food and Drug Administration (FDA) for clinical treatment (148). However, the necessity for high-quality randomized controlled clinical trials persists to validate its efficacy and assess associated risks in future applications. Pharmacotherapy remains an important integral part of the treatment of SCI-induced NP due to its high compliance and relatively small financial burden (Figure 2).

## 4 Non-pharmacological therapeutic interventions of pathologic neuropathic pain following SCI

### 4.1 Electroacupuncture stimulation for post-spinal cord injury neuropathic pain

Acupuncture, a traditional Chinese medicine treatment method with over three thousand years of practical experience, has been utilized in the management of various diseases (149). Electroacupuncture (EA) is a specific acupuncture technique that has demonstrated efficacy in treating numerous SCI-related conditions, such as dyskinesia, NP, and spasticity (150). However, the precise mechanism underlying electroacupuncture’s effectiveness in alleviating NP remains unclear and warrants further investigation. Liu et al. explore the effect that electroacupuncture treatment in SCI rats activating miR-214,which targeted pain related protein Nav1.3, may reduce NP after SCI Given its non-invasive nature, safety profile, affordability, and minimal side effects, electroacupuncture holds significant promise in managing NP post-spinal cord injury.

### 4.2 Non-invasive stimulation techniques for post-spinal cord injury neuropathic pain

Repetitive transcranial magnetic stimulation (rTMS) is a non-invasive neuromodulation technique that utilizes electromagnetic coils to generate magnetic fields, forming pulse stimuli in the cerebral cortex and even deep brain regions, thereby regulating cortical excitability and promoting functional remodeling (151). In published studies, the most common stimulation site for NP following SCI with rTMS is the M1 region, and the most commonly used dose is 5–10 Hz, which has been shown to produce the best analgesic effects after 5–10 treatments (152). In a randomized double-blind controlled study of rTMS for acute NP (pain duration ≤3 weeks) after SCI, 48 patients were randomly divided into a high-frequency rTMS group (rTMS stimulation frequency of 10 Hz, stimulation site of M1 contralateral to the affected hand, once a day, for a total of 18 sessions) and a sham stimulation group. No analgesic drugs (such as gabapentin, pregabalin, etc.) were taken during the treatment period, and the results showed that the high-frequency rTMS group had significantly reduced pain after treatment compared with the sham stimulation group (153). Other studies have also investigated the effects of 10 Hz rTMS over the dorsolateral left prefrontal cortex (DLPFC) region (once a day for a total of 10 treatments over a 2-week period) on NP following SCI (154). Results showed a significant decrease in daily pain scores during stimulation in the high-frequency rTMS group but not in the sham group. Although the target areas stimulated in the rTMS studies were different, they all resulted in significant pain reduction for the patients. Regarding the mechanism of action, transcranial magnetic stimulation seem reduce pro-inflammatory cytokines, such as IL-1b, IL-6, and TNF-a, while increasing anti-inflammatory cytokines, including IL-10 and brain-derived neurotrophic factor (BDNF), in cortical and subcortical tissues (152); additional *in vitro* and *in vivo* studies and clinical trials are required to investigate these mechanism. This inhibits neuroinflammation after SCI, consequently reducing NP.

Transcranial direct current stimulation (tDCS) is a non-invasive technique that employs a constant, low-intensity direct current (typically ranging from 1 to 2 mA) to modulate neuronal activity in the cerebral cortex (155). Research has explored the application of tDCS, specifically with a single current intensity of 2 mA, on NP patients following SCI. Assessment was conducted after 20 min of continuous treatment, revealing significantly lower pain scores in the tDCS group compared to the sham stimulation group immediately post-treatment and 24 h later. This suggests that the analgesic effect of single tDCS extends beyond the stimulation period, manifesting as an aftereffect well beyond the duration of stimulation (156). In a randomized controlled trial, the efficacy of tDCS on NP post-SCI was evaluated in two treatment phases separated by a 3-month interval. In the initial stage, 33 patients were randomly assigned to either the tDCS group (*n* = 16) or the sham stimulation group (*n* = 17). The treatment involved administering tDCS at a 2 mA DC intensity, once every 20 min, once daily, for a total of 5 days. In the subsequent phase (comprising 9 patients in total, 6 in the tDCS group and 3 in the sham stimulation group), a further 10 tDCS sessions (one session per day lasting 20 min) were administered. Results demonstrated a significant reduction in pain scores in the tDCS group compared to the sham group at the 1-week follow-up in the first phase and at the 4-week follow-up in the second phase (157). The most widely proposed mechanism underlying the effectiveness of tDCS for NP following SCI involves the modulation of spontaneous cortical neuronal activity through polarized resting membranes. This modulation affects various pain-related structures, including the anterior cingulate gyrus and periaqueductal gray, ultimately modulating the affective components of pain perception and experience (27). In summary, both rTMS and tDCS have demonstrated analgesic effects on NP following SCI. However, limited clinical data and studies exist, necessitating further research to elucidate their effects and mechanisms of action.

### 4.3 Invasive stimulation techniques for post-spinal cord injury neuropathic pain

**Figure 3:**
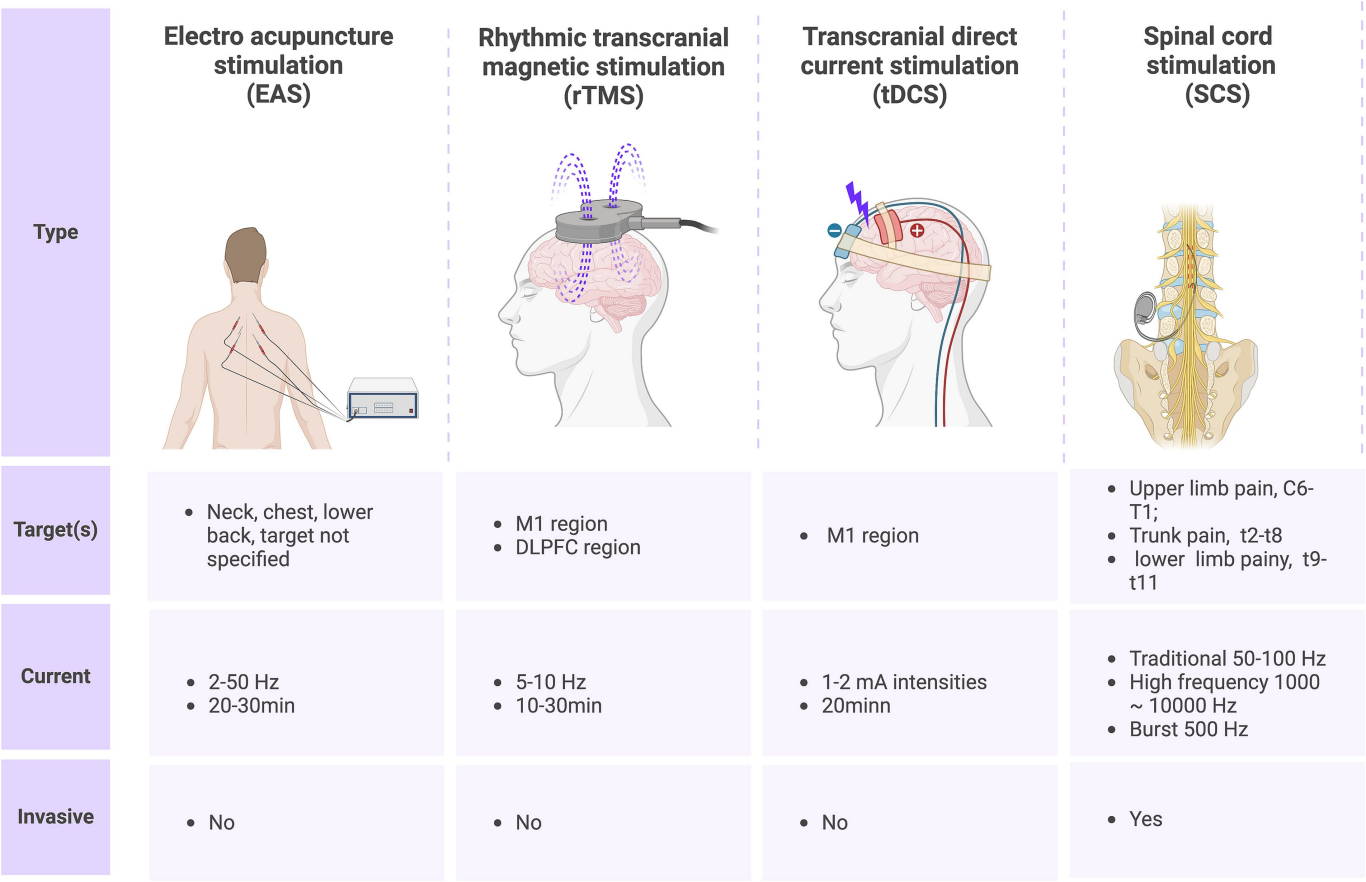
Current nonpharmacological treatments for SCI-induced neuropathic pain and their characteristics.

Spinal cord stimulation (SCS) was initially proposed based on Melzack and Wall’s gating theory, which posits the existence of a “gate action” mechanism within the spinal cord and brain’s pain conduction pathways. According to this theory, afferent impulses from Aδ and C fibers open the gate, allowing pain signals to be transmitted to the central nervous system and produce the sensation of pain. Conversely, when SCS stimulates Aβ fibers, the gate closes, resulting in retrograde inhibition of nociceptive signals entering the spinal cord, thus achieving analgesia (158). Subsequent research has revealed that SCS not only activates brainstem nuclei and the rostral ventromedial medulla oblongata but also modulates nociceptive signals at the spinal cord level through descending fiber projections from supraspinal cell regions (159). Furthermore, SCS can activate the frontal gyrus, limbic system, and thalamus via the pain ascending conduction pathway, thereby exerting analgesic effects and improving cognitive function (160). Spinal cord electrical stimulation has been widely utilized for various refractory pain conditions. This procedure involves identifying the corresponding spinal cord segments of pain under local anesthesia and implanting electrodes into the spinal epidural space to deliver pulse currents and stimulate the spinal cord nerves (28). The post-operative spinal cord electrical stimulation typically occurs in two phases: a test phase and a permanent implant phase. During the test phase, a trial system is implanted for approximately one week to assess its effectiveness. If there is a 50% or greater improvement in pain baseline values, the permanent implant system is then implanted (161). SCS has been found to be more effective in improving pain in patients with incomplete SCI compared to those with complete SCI. This efficacy may be influenced by factors such as the distance between the injury site and the implanted SCS electrode, as well as the number of residual intact nerve fibers (28). With advancements in technology, various new modes of SCS have emerged. One such mode is burst SCS, which is characterized by low-energy delivery. Burst SCS applies five continuous wave pulse sequences at specific internal frequencies (500 Hz) and pulse widths (1 ms, with intervals of 1 ms), occurring 40 times per second. Burst SCS has been shown to effectively inhibit pain below the level of injury in patients with complete paraplegia. A case report demonstrates a significant reduction in the frequency and intensity of pain, with therapeutic effects lasting over three months (162). However, extensive clinical trials are still required to substantiate these findings. The other is high-frequency SCS therapy, which provides electrical stimulation pulses of short duration (30 μs) and high frequency (10,000 Hz) without paresthesia compared with traditional spinal cord stimulation. A sham-controlled study showed that both high-frequency SCS and burst SCS could reduce levels of SCI-related NP (163). In summary, for patients with NP after spinal cord injury, non-pharmacological treatment has different characteristics (Figure 3), but all of them have great potential in reducing pain and improving quality of life.

## 5 Future and prospect of NP treatment after SCI

Despite the variety of treatments available for SCI, current options remain insufficient to fully address the associated clinical problems, and many patients continue to experience persistent pain. Recent research has increasingly focused on the role of gut microbiota in SCI. Chen et al. analyzed the relationship between gut microbiota, inflammatory markers in serum, and pain behavior parameters. Their findings revealed a significant increase in the abundance of *Helicobacter pylori*, Bacillus spp., Streptococcus spp., Roche spp., and Lactobacillus spp., while genera such as Ignacillus spp., Butyric monas spp., and Escherichia spp. showed reduced abundance. Another study highlighted that oral antibiotics could induce changes in gut microbiota that mitigate the development of NP, accompanied by improvements in inflammatory parameters. This suggests that gut microbiota might influence the development and progression of NP, potentially through the modulation of pro-inflammatory and anti-inflammatory T cells (164). These findings could offer new avenues for studying NP following SCI. Additionally, NP after SCI is often linked with psychological factors such as fatigue, anxiety, and depression. In this context, mental imagery (MI) therapy has garnered increasing interest. Research indicates that MI can effectively alleviate NP in SCI patients (165). This method is noted for its simplicity, safety, and reproducibility; however, its efficacy can vary among patients. While most studies report that MI significantly relieves NP after SCI (166, 167), some research suggests that MI may be less effective or even exacerbate pain in certain cases (168, 169). These discrepancies may stem from differences in SCI severity, timing, and location of injury, or variations in MI treatment protocols. Therefore, further investigation is necessary to clarify its efficacy and underlying mechanisms. Nanomedicine represents an emerging field within nanotechnology, characterized by unique biological properties such as a high surface-to-volume ratio, distinctive structural attributes, capacity for surface modification, ability to permeate biological barriers, and extended circulation time in the bloodstream (170). The utilization of nanomedicines can significantly enhance the pharmacokinetic and pharmacodynamic profiles of drugs, enable prolonged release, and achieve targeted delivery to specific sites through labeling with selective ligands. In the realm of cell therapy, nanomedicine plays a pivotal role by efficiently guiding cell differentiation and trans differentiation, and mitigating immunogenic responses associated with cell therapy through the encapsulation of various proteins and polymers. In gene therapy, nanomaterials serve as proficient vectors for delivering different genes, thereby addressing the limitations inherent in both viral and non-viral vectors (171). Hence, nanomedicine enables targeted drug delivery for neuropathic pain (NP) following spinal cord injury (SCI), enhancing therapeutic efficacy and minimizing side effects. Furthermore, it facilitates the orientation of reparative cells, biological factors, and genes, thereby advancing spinal cord regeneration and alleviating pain. Nanomaterials offer a novel and promising vehicle for the treatment of NP following SCI, demonstrating substantial potential for future development. In clinical practice, it is essential to differentiate patients based on factors such as symptomatology, pain levels, complications, gender, and age. Tailoring treatment plans to these individual differences, and employing a combination of therapeutic approaches, may lead to optimal outcomes.

## 6 Conclusion

Neuropathic pain is a prevalent complication of SCI, described by patients as an intolerable condition that significantly impacts their long-term quality of life. It emerges as a consequence of neuronal remodeling subsequent to SCI, representing a pathological response triggered by neurological injury induced by local mechanical compression, ischemia, and inflammation of the spinal cord. The pathogenic mechanisms underlying NP following SCI are intricate, with most studies focusing on rodent models. This review comprehensively summarizes current research on the mechanisms underlying spinal cord injury (SCI)-related neuropathic pain (NP). At the peripheral level, it discusses nociceptor hyperexcitability and spontaneous activity. At the spinal cord level, it addresses changes in neuronal excitability, glial cell activation, calcium channel expression, immune-inflammatory responses, neurotransmitter secretion disorders, neurotrophic factor imbalances, and the role of non-coding RNAs. Furthermore, at the supraspinal level, it explores structural plasticity changes in brain regions and alterations in brain metabolism.

In clinical practice, the primary treatments for SCI-related NP typically involve analgesic, anticonvulsant, antidepressant, and antispasmodic medications. However, their effectiveness is often limited, and they may elicit adverse reactions that do not fully address the needs of patients. Emerging therapies for SCI-NP include novel drugs such as natural compounds and antisense oligonucleotides, which offer advantages such as minimal side effects, easy access to raw materials, and adaptability. Nonetheless, their established therapeutic efficacy requires validation through additional clinical trials.

Electroacupuncture, a therapeutic modality in traditional Chinese medicine, plays a significant role in the management of SCI-NP. Particularly beneficial for patients unable to undergo conventional medical treatment, electroacupuncture offers fewer side effects and enhanced therapeutic outcomes. Furthermore, it can be integrated with other treatments to potentially reduce dosage requirements and achieve a synergistic therapeutic effect.

Both rTMS and tDCS are non-invasive brain stimulation techniques that have shown promise in reducing NP following SCI, thereby improving patients’ quality of life. However, the lack of a definitive and standardized treatment regimen for either rTMS or tDCS, along with the heterogeneity of existing studies in terms of intervention cycles and outcome measures, complicates comparisons between interventions and anticipated outcomes. This lack of consensus in clinical application results in variable treatment outcomes. While spinal cord stimulation holds promise for treating NP after SCI, research in this area is still in its developmental stages. Practical limitations such as cost, intervention protocols, treatment frequency, and technical variability, as well as ethical considerations regarding the use of sham interventions, hinder its widespread adoption. Further research is warranted to establish its precise efficacy. In cases where patients exhibit poor responsiveness to conventional and non-invasive treatments, early consideration of spinal cord stimulation intervention may be beneficial in delaying pain progression and improving overall symptoms and quality of life.

In conclusion, the intricate mechanism underlying NP after SCI underscores the need for individualized treatment plans tailored to each patient’s unique condition. Optimal outcomes are likely to be achieved through a combination of various treatment modalities.
